# Operational research as a mechanism to improve treatment outcomes for drug-resistant TB in the WHO European Region

**DOI:** 10.5588/ijtldopen.24.0035

**Published:** 2024-03-01

**Authors:** G.B. Migliori, O. Korotych, J. Achar, A. Ciobanu, G. Dravniece, M. Germanovych, E. Gurbanova, A. Hovhannesyan, N. Khachatryan, L. Kuksa, N. Lomtadze, M.L. Rich, A. Skrahina, A. Yedilbayev

**Affiliations:** ^1^ Servizio di Epidemiologia Clinica delle Malattie Respiratorie, Istituti Clinici Scientifici Maugeri, Istituto di Ricovero e Cura a Carattere Scientifico, Tradate, Italy;; ^2^ Joint Infectious Diseases Unit, World Health Organization Regional Office for Europe, Copenhagen, Denmark;; ^3^ Department of Global Public Health, Karolinska Institutet, Stockholm, Sweden;; ^4^ Department of Science and Innovation–National Research Foundation Centre of Excellence for Biomedical Tuberculosis Research, South Africa Medical Research Council Centre for Tuberculosis Research, Division of Molecular Biology and Human Genetics, Faculty of Medicine and Health Sciences, Stellenbosch University, Cape Town, South Africa;; ^5^ PATH, Kyiv, Ukraine;; ^6^ Lung Clinic, University of Tartu, Tartu, Estonia;; ^7^ National Center of Pulmonology, Ministry of Health, Abovyan, Armenia;; ^8^ Tuberculosis and Lung Disease Clinic, Riga East Univesity Hospital, Riga, Latvia;; ^9^ National Center for Tuberculosis and Lung Diseases, Tbilisi, Georgia;; ^10^ The University of Georgia, Tbilisi, Georgia,; 11 Division of Global Health Equity, Brigham and Women’s Hospital, Boston, MA, USA;; ^12^ Republican Scientific and Practical Centre for Pulmonology and Tuberculosis, Minsk, Belarus

**Keywords:** tuberculosis, MDR-TB, shorter regimens, rifampicin resistant, mSTR

## Abstract

In 2022, the WHO European Region accounted for 15.1% of all incident rifampicin-resistant/multidrug-resistant TB (RR/MDR-TB) cases. Most occurred in 18 high-priority countries of eastern Europe and central Asia, many of which joined an initiative led by the WHO Regional Office for Europe. The aim was to introduce three, fully oral, 9-month modified shorter treatment regimens (mSTR) to treat RR/MDR-TB under operational research conditions. The three regimens were: 1) bedaquiline + linezolid + levofloxacin + clofazimine + cycloserine (BdqLzdLfxCfzCs); 2) BdqLzdLfxCfz + delamanid (Dlm) for children over 6 years of age and adults; and 3) DlmLzdLfxCfz for children under 6 years of age. The project aimed to enhance treatment success, facilitate mSTR implementation, promote quality of care and build research capacity, while also contributing to global knowledge on all-oral mSTR use. Between April 2020 and June 2022, >2,800 patients underwent mSTR treatment in the WHO European Region. This unique experience promoted further collaboration with national tuberculosis programmes, health authorities, experts and donors within and outside Europe, with a focus on implementing operational research and improving the quality of care in high TB burden countries of the region. In the hope of encouraging others to adopt this model, we have described the principles of the initiative, its strengths and weaknesses and next steps.

## RÉSUMÉ

En 2022, la Région européenne de l'OMS a recensé 15,1% de l'ensemble des cas de TB résistante à la rifampicine/multirésistante aux médicaments (RR/MDR-TB). La majorité de ces cas ont eu lieu dans 18 pays hautement prioritaires d'Europe orientale et d'Asie centrale, parmi lesquels de nombreux ont adhéré à une initiative dirigée par le Bureau régional de l'OMS pour l'Europe. L'objectif était de mettre en place trois schémas thérapeutiques modifiés plus courts de 9 mois, entièrement oraux, (mSTR, pour l’anglais “*fully oral, 9-month modified shorter treatment regimens* ») pour le traitement de la RR/MDR-TB dans le cadre d'une recherche opérationnelle. Ces trois schémas étaient les suivants 1) bédaquiline + linézolide + lévofloxacine + clofazimine + cyclosérine (BdqLzdLfxCfzCs) ; 2) BdqLzdLfxCfz + delamanid (Dlm) pour les enfants de plus de 6 ans et les adultes ; et 3) DlmLzdLfxCfz pour les enfants de moins de 6 ans. Le projet visait à améliorer l'efficacité des traitements, à faciliter l'application des mSTR, à promouvoir la qualité des soins et à renforcer les capacités de recherche, tout en contribuant aux connaissances mondiales sur l'utilisation des mSTR par voie orale. Entre avril 2020 et juin 2022, plus de 2 800 patients ont reçu un traitement par mSTR dans la Région Européenne de l'OMS. Cette expérience unique a encouragé la continuation de la collaboration avec les programmes nationaux de lutte contre la TB, les autorités sanitaires, les experts et les donateurs tant en Europe qu'à l'étranger. L'accent est mis sur la mise en œuvre de la recherche opérationnelle et l'amélioration de la qualité des soins dans les pays de la région où la TB est fortement prévalente. Nous avons détaillé les principes de l'initiative, ses avantages et ses inconvénients, dans l'espoir d'inciter d'autres pays à suivre cet exemple, tout en exposant les étapes à venir.

In 2022, the WHO estimated that about 410,000 rifampicin resistant (RR)/multidrug-resistant TB (MDR-TB) cases occurred globally, which constitutes 3.9% of the global TB burden.^1^ The proportion of RR/MDR-TB was 3.3% among new and 17% among previously treated cases.^1^ Although the WHO European Region accounted for only 2.2% of the global TB burden (the regional incidence rate per 100,000 population was 25 against 208 globally), it had 15.1% of all incident RR/MDR-TB cases. The estimated prevalence of RR/MDR-TB was 24% among new and 54% among previously treated cases.^1^ The management of RR/MDR-TB patients is clinically challenging due to the complexity of its diagnosis, the long treatment duration, frequency of adverse events and the higher cost of second-line drugs.^2^

**Figure 1.**
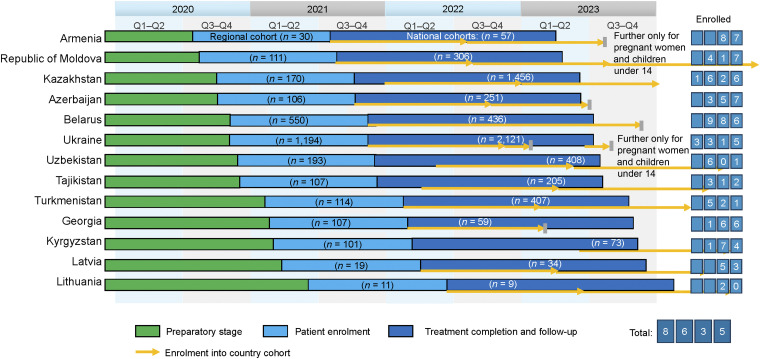
Project timeline and enrolment number by country. Q = quarter.

In the WHO European Region, approximately half of incident RR/MDR-TB cases are diagnosed: with 34,630 notified cases vs. an estimated number of 61,500. Most (85%) incident TB cases occur in 18 countries of eastern Europe and central Asia, which are referred to as high-priority countries^3^ in the WHO European Region.^4^ Of these 18 high-priority countries, 13 (Armenia, Azerbaijan, Belarus, Georgia, Kazakhstan, Kyrgyzstan, Latvia, Lithuania, Republic of Moldova, Tajikistan, Turkmenistan, Ukraine and Uzbekistan) joined the WHO European Regional initiative to introduce a fully oral, modified shorter treatment regimen (mSTR) for RR/MDR-TB under operational research (OR) conditions (Figure 1). In 2019 (prior to the study), the combined treatment success rate for RR/MDR-TB (excluding pre-extensively drug-resistant TB [pre-XDR-TB] and XDR-TB) was 68.6%, which is significantly lower than the 80% regional milestone for 2025 according to the ‘Tuberculosis Action Plan for the WHO European Region 2023–2030’.^5^ Given the epidemiological situation in the region, the availability of new tools and approaches in prevention, diagnosis and treatment of TB and drug-resistant TB (DR-TB), development and implementation of breakthrough interventions are required to accelerate the progress in Europe, with specific focus on countries with the highest burden of disease.

## IMPORTANCE OF OPERATIONAL RESEARCH AND THE NEED FOR IMPROVED QUALITY OF CARE

In the WHO strategies for TB prevention and care,^6^ research – including OR – initially appeared as the sixth element of the Stop TB Strategy (2006–2015) before evolving into a dedicated third pillar in the End TB Strategy 2016–2035.^1,7^ There are many links between research and the broader aims of TB prevention and care. We clearly require new vaccines, diagnostics, drugs/regimens and ongoing advocacy efforts, as evidenced by the initiative to raise at least US$22 billion for research by 2027, launched during the 2023 United Nations High-Level meeting.^1,8^ The ‘Tuberculosis Action Plan for the WHO European Region 2023–2030’^5^ urges Member States to ensure universal access to prevention, diagnosis and treatment for both drug-susceptible (DS-) and DR-TB. Evidence-based practices and high-quality services must be universally available, accessible, affordable and culturally sensitive. Because healthcare is a fundamental right, Member States are urged to uphold principles of equity, ethics, gender equality and human rights. As research plays a crucial role in achieving universal access, Member States are advised to enhance research infrastructure, establish legislative frameworks for swift adoption of innovations, and develop and fund national TB research plans to facilitate their implementation.

**Figure 2.**
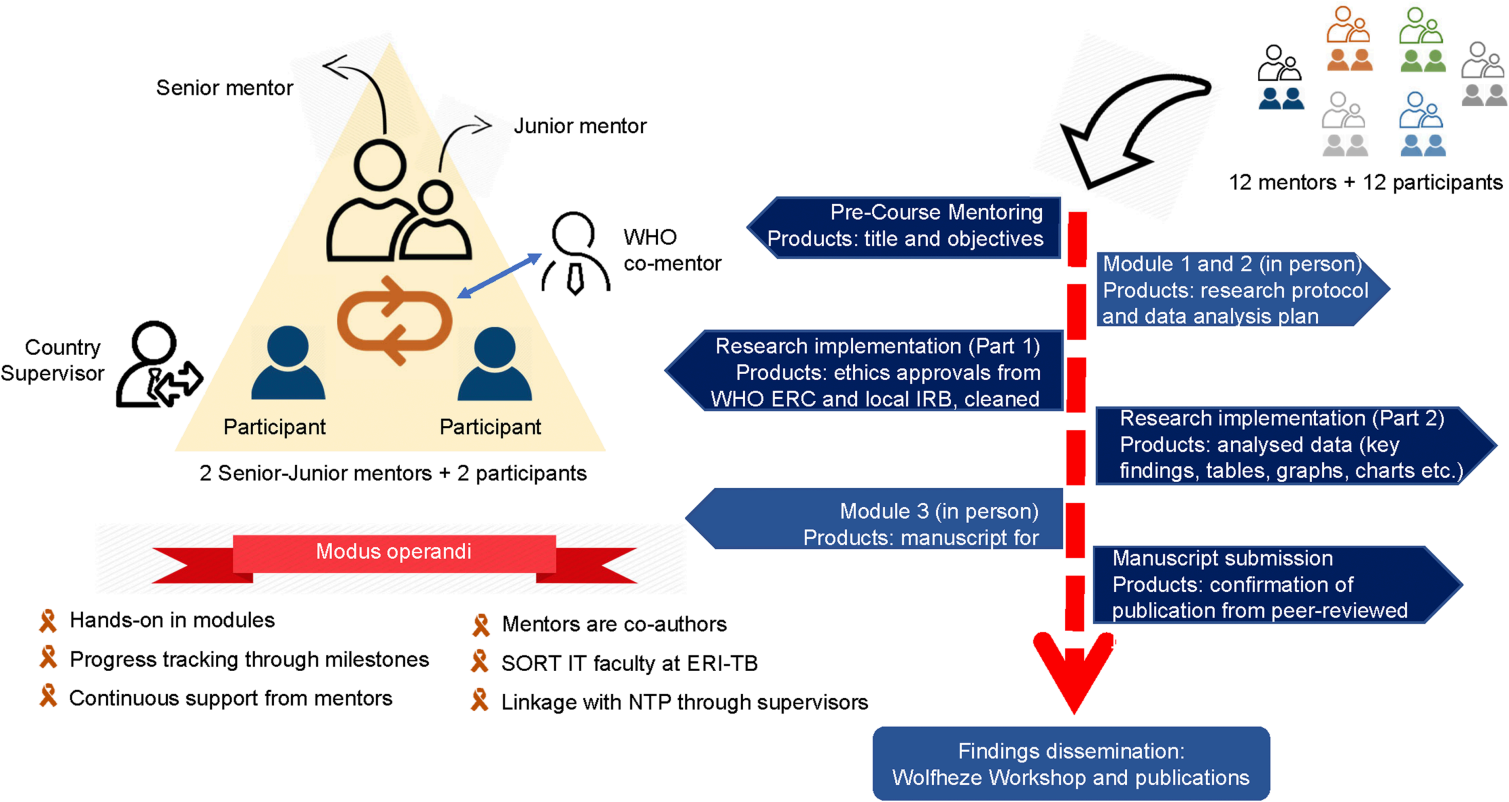
SORT-TB mentorship model and study process. IRB = Institutional Review Board; SORT IT = Structured Operational Research and Training IniTiative; ERI-TB = European TB Research Initiative; NTP = national TB programme.

However, research is more than this, as it involves dissemination (e.g., when scientific information is not published, it is lost as not traceable/promptly available to the scientific community) and serves as a repository of knowledge. Importantly, research is the cornerstone of evidence-based medicine, forming the basis for quality guidelines (following the Grading of Recommendations Assessment, Development and Evaluation [GRADE] methodology) and policy development. As such, it is a fundamental tool for enhancing the clinical management of patients. Specifically, OR expedites, boosts and supports the programmatic implementation of innovations in the field of TB prevention, diagnosis and care. Furthermore, OR promotes the uptake of recommendations and improvements in the quality of clinical care is garnering increasing attention. The WHO is actively assisting national tuberculosis programmes (NTPs) in incorporating a meticulously budgeted OR plan into their national strategic plans. This aims to guarantee the identification of key research areas at the national programme level and strives to establish a proficient team equipped with both the necessary capacity and funding to spearhead and execute these research initiatives effectively.^9,10^ In alignment with the Tuberculosis Action Plan for the WHO European Region, 2016–2020, the WHO Regional Office for Europe established the European TB Research Initiative (ERI-TB) in 2016. The regional platform aims to achieve several objectives, including mapping ongoing TB-related research, developing and updating a regional research priority agenda, ensuring the engagement of civil society, facilitating the dissemination of research results and translating these into evidence-based policies and practices. Additionally, ERI-TB is committed to identifying and facilitating measures to address funding gaps in TB research within the European Region.^11^ As one of the initial steps, ERI-TB conducted a study in 2017 to identify research gaps in the region and design the regional TB research agenda (ERA-TB). The study identified 76 research questions, of which 20 were given the highest priority.^12^ As of 2024, the ERI-TB Secretariat, hosted at the WHO Regional Office for Europe (Copenhagen, Denmark) is in the process of revising ERA-TB to align it with the goals and targets of the TB action plan.^5^ Furthermore, ERI-TB launched Structured Operational Research Training on TB (SORT-TB) in 2018 to boost implementation of ERA-TB by engaging young researchers and programmatic specialists in the implementation of research with the use of programmatically collected data. SORT-TB adapted a mentorship model (Figure 2), under which young researchers work on their projects under the supervision of experienced scientists and TB professionals. The two cohorts of SORT-TB resulted in 25 publications in peer-reviewed journals. However, a huge gap in funding and capacity exists in this area,^1,13,14^ and regional experiences focused on developing both research and clinical management capacity for the benefit of DS- and DR-TB patients were not available.

## REGIONAL OPERATIONAL RESEARCH INITIATIVE ON MSTR

The introduction of the shorter all-oral, bedaquiline (Bdq) containing, 9-month regimen (STR; including Bdq, moxifloxacin [Mfx], prothionamide [Pto], clofazimine [Cfz], pyrazinamide [Pza], high-dose isoniazid [Inh^H^] and ethambutol [Emb]) was first recommended in the 2020 WHO consolidated guidelines on TB.^15^ However, studies from several settings, including eastern Europe, demonstrated frequent resistance to one or more constituent drugs, a contra-indication to the regimen’s use.^16–19^ The prevalence of resistance in Europe and Latin America exceeded 50–60% for the first-line drugs (Emb and Pza), 50% for Pto and 40% for fluoroquinolones and kanamycin (although the latter is no longer prioritised for use in MDR/RR-TB regimens).^19^ Furthermore, patient preference, limited evidence of effectiveness and reduced implementation complexity supported a shift towards injectable-free regimens. In 2020, WHO guidelines advised the use of OR to modify the standard short, all-oral treatment regimen for MDR/RR-TB in settings with high prevalence of resistance to the first-line drugs. The guidelines stipulated that any modifications to STR, in which one or more drugs are replaced may be introduced in the context of OR to evaluate their effectiveness safety and tolerability.^15^ Thus, as part of the transition to the latest WHO policy guidance on DR-TB, the WHO Regional Office for Europe (via the ERI-TB) launched a regional OR project on the introduction of mSTR for MDR/RR-TB in collaboration with NTPs and with the support from the Global Fund to Fight AIDS, TB and Malaria, the United States Agency for International Development and the German Government. The goals of the mSTR OR initiative were 1) to enhance treatment success: improve treatment success rates for MDR/RR-TB in the participating countries through targeted interventions; 2) to facilitate mSTR implementation: streamline the introduction of mSTR for patients with MDR/RR-TB under OR conditions, ensuring a smooth integration into existing healthcare frameworks; 3) to promote quality clinical care: foster the delivery of high-quality clinical care for patients with MDR/RR-TB through the insights gained from OR, enhancing overall patient outcomes; 4) to build research capacity: strengthen research capacity in participating countries, empowering local stakeholders with the skills and knowledge needed to conduct effective OR and; 5) to contribute to global knowledge: contribute to the understanding of the effectiveness and safety of all-oral mSTR for MDR/RR-TB, generating valuable insights that can inform future practices and policies.

Leveraging insights gained from evidence presented to the WHO Guideline Development Group on DR-TB treatment (particularly, the data available from South Africa on the use of a 9-month Bdq-containing regimen), in September 2019, the WHO Regional Office for Europe took decisive action. A Task Force was established to craft a feasible modification to this regimen, specifically tailored to the prevalent resistance patterns and the region’s needs. The following objectives were delineated: 1) develop a comprehensive package for regional OR encompassing a standardised set of materials and tools; 2) facilitate OR implementation at the country level, ensuring practicality and effectiveness of the modified regimen in diverse healthcare settings; 3) harmonise data collection methods and analyse the gathered information systematically, promoting consistency and comparability across different settings; and 4) generate high-quality evidence with the aim of providing robust data for submission to the forthcoming session of the WHO Guideline Development Group on DR-TB treatment. Subsequently, the Regional Office has been providing technical support to Member States who joined the regional initiative through its Task Force, including guidance on the country-specific adaptation of the study package, training for country study teams, support for ethical approvals and guidance on the use of the data collection tool and monitoring of the implementation progress while ensuring the completeness and quality of the collected data.

## DEVELOPING THE PROTOCOL AND FIRST STEPS

The regional OR package, which included a research protocol, case report forms (CRFs), a patient informed consent form and educational materials, guide for management of adverse events, an active drugs safety monitoring (aDSM) package, and database and data collection instructions, was first presented during a regional meeting in Kyiv, Ukraine, on 10–11 December 2019, followed by a period of country adaptation. The master protocol of the regional OR on the introduction of mSTR for MDR/RR-TB was approved by the WHO Ethics Review Committee (ERC) at the beginning of July 2020 and subsequently, all the countries involved received both national and WHO ERC approvals.

Three 9-month treatment regimens were chosen by the task force experts to be implemented in the WHO European Region: 1) Bdq, linezolid (Lzd), levofloxacin (Lvx), clofazimine (Cfz) and cycloserine (Cs); 2) BdqLzdLvxCfz and delamanid (Dlm) for children aged >6 years and adults; and 3) DlmLzdLvxCfz for children aged <6 years. The second regimen was used in patients with suspected resistance or intolerance to Cs. All patients with evidence of resistance to at least rifampicin (Rif) by phenotypic or rapid molecular drug susceptibility testing were invited to participate in the OR project if they were willing and able to give informed consent to be enrolled in the research project and for follow-up; had bacteriologically confirmed TB with initial laboratory result of resistance to at least Rif; children with clinically diagnosed RR-TB based on history of close contact with a confirmed RR-TB case (with no evidence of resistance or confirmed susceptibility to medicines composing the mSTR).

Patients with any of the following criteria were not eligible for inclusion: those unable to take oral medication, those taking any medications contraindicated for medications included in the mSTRs; those with known allergy or hypersensitivity to any of the drugs in the mSTR regimens; those with documented resistance to a fluoroquinolone or other components of mSTR regimen with reliable drug susceptibility testing (Bdq, Dlm, Lzd, Cfz); those who have been exposed to TB treatment with drugs from the mSTR regimens for ≥1 month;^*^ those with tuberculous meningitis, miliary TB or tuberculous osteomyelitis; those with a heart rate-corrected QT (QTc with Fridericia correction) interval of ≥500 msec on electrocardiogram at screening despite correction of serum electrolytes; those with liver enzymes >3 times the upper limit of normal; those with creatinine clearance below 30 mL/min per 1.73 m^2^ body surface area; patients in a very severe clinical condition (Karnofski scale <40 or ECOG = 4).

**Figure 3.**
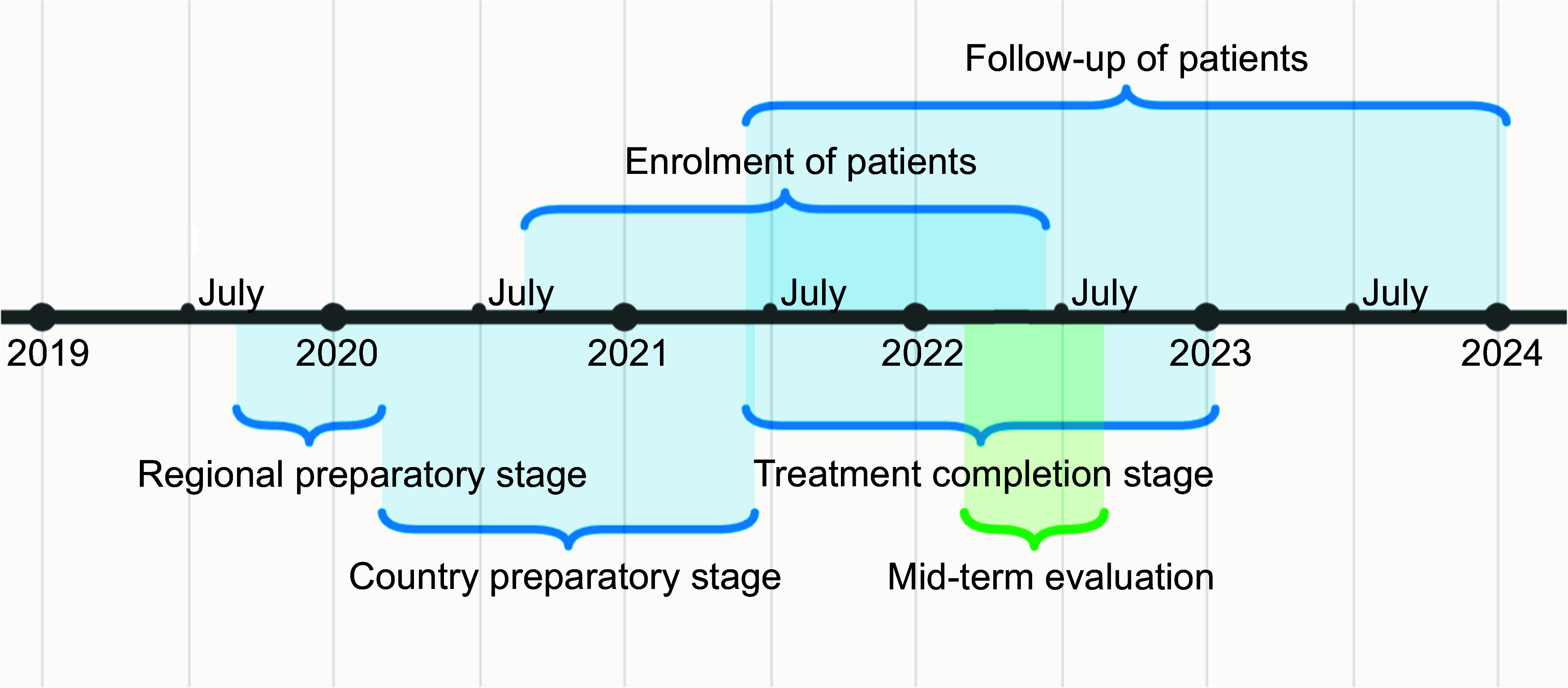
Regional cohort timeline.

The project timeline is summarised in Figure 3. The main objectives of the study were to determine the following: 1) end of treatment outcomes; 2) safety of mSTR regimens – in particular, the rate, time to onset and severity of serious adverse events (SAEs) and adverse events of interest (AEI) of grade 3 and higher; 3) time to sputum culture conversion; 4) frequency of resistance acquisition to mSTR components; and 5) TB recurrence rate within 12 months of successful completion of treatment. Following a model similar to the SORT-TB mentorship approach, the mSTR Task Force appointed two experts for each country. These experts assumed roles of oversight, training, supervision and monitoring throughout the implementation process. To kickstart the initiative, initial training sessions in each country were organised for national-level teams. These sessions served as forums for detailed discussions on various facets of research implementation, aiming to enhance the preparation of each country-specific research protocol. The engagement of dedicated experts and comprehensive training underscored the commitment to ensuring the successful and standardised execution of mSTR activities across diverse national contexts. The agenda of training sessions for participating facilities included practical information on the research protocol with inclusion and exclusion criteria, enrolment procedures, diagnosis and management of AIE/SAE, treatment monitoring and aDSM, electrocardiogram reading and neurologic examinations, specifically the screening for peripheral neuropathy and visual acuity. Finally, training on the paper and MS Excel-based (Microsoft, Redmond, WA, USA) CRFs and Epi Info v7 software (Centers for Disease Control and Prevention, Atlanta, GA, USA) for research data entry was also conducted in practical sessions, with an overall impact on the countries’ capacities to manage RR/MDR-TB cases. Subsequently, as part of the preparations for mSTR OR implementation, the national teams arranged and conducted training workshops for physicians and other healthcare professionals providing services to patients in the facilities where the research was carried out. Before or during the preparation of the research, training on clinical management aspects was organised for clinicians at regional institutions and for district physicians/TB physicians.

As part of the research, each country was committed to recruiting patients into the regional research cohort within 12 months from the date of patient enrolment in the country in question. The challenges, which were successfully tackled, were to implement the study within the infrastructure of the NTP and within the time devoted by staff to clinical activities, and conduct the study during the COVID-19 pandemic despite travel difficulties and the extra effort required by clinicians.^20–23^ The first patients were enrolled in August 2020 in Armenia. All of the 13 WHO European Region countries mentioned above joined the initiative and initiated patient enrolment by June 2021 (Figure 2). By June 2022, there were more than 2,800 patients undergoing mSTR treatment in the regional cohort. Country cohorts were also established in all 13 countries after enrolment in the regional cohort was completed, with NTPs continuing to provide patients with access to mSTR regimens in OR settings. As of December 2023, more than 5,800 patients were enrolled in country cohorts.

The implementation of the regional and the country cohorts was constantly monitored, with quarterly supervision missions (in person or internet-based) and with periodic meetings. In total, more than 170 missions were conducted by the mSTR task force experts between March 2020 and December 2023. The annual meetings were conducted on 25–26 August 2022 by the WHO Regional Office for Europe virtually (the first annual meeting of the mSTR national study teams from the 13 countries involved) and on 19 November 2023 in Paris, France. In parallel the WHO Regional Office for Europe focused on building capacity for data analysis and dissemination of results. Three workshops were carried out in October 2022, July 2023 and November 2023 to provide national study teams with the set of skills to clean, validate and analyse mSTR data, as well as to write abstracts and research manuscripts to better understand and disseminate the findings.

## IMPROVING QUALITY OF CARE

The goals of the mSTR OR were to promote high-quality clinical care for patients with DR-TB, including adequate diagnosis and treatment monitoring, aDSM and scale-up of outpatient models of care, specifically video-supported treatment. Therefore, in September 2020, to ensure clinical support to countries during the enrolment procedure (particularly for complicated cases) and provide advice on treatment (e.g., regimen modification, treatment discontinuation, dose reduction) and, overall, improve clinical care, the WHO Regional Office for Europe, with support from the Center for Health Policies and Studies (PAS Center) within the regional TB REP 2.0 Project established the VMC for mSTR. The VMC plays a crucial advisory role by offering expert opinions and proposing best possible treatment options for the case management of complex clinical cases. However, the ultimate responsibility for the final decisions lies with individual clinicians and/or national consilia. This collaborative approach ensures that medical decisions are well-informed, taking into account both expert guidance and the unique aspects of each clinical situation. Between 2020 and 2023, the VMC (which operates in English and Russian), supported clinical questions regarding more than 400 patients from the 13 countries, responding within an average time of 39 hours. The main reasons to consult the VMC were enrolment procedures, end-of-treatment decisions for patients undergoing mSTR, clinical management of complicated cases, management of AEs and co-infections.

The VMC, as well as other similar initiatives (e.g., the Global TB Network Consilium)^24^ offers a rapid, accessible, free-cost online tool for clinicians called to manage difficult-to-treat cases of TB, contributing to a better understanding of existing WHO guidelines^24,25^ and improved clinical management of TB patients in the Region. Furthermore, the VMC organised regular webinars aiming to facilitate timely dissemination of TB developments and best practices, to engage with global experts and to promote cross-country knowledge exchange. Almost 2,000 unique participants took advantage, from 25 webinars held during the VMC life span.

Overall, the countries participating in the mSTR OR achieved improved clinical care resulting from, among others, training, regular supervision, the possibility to ensure rapid molecular diagnosis implementing Xpert^®^ MTB/XDR (Cepheid, Sunnyvale, CA, USA) and to implement quality treatment monitoring (with access to clinical and biochemical tests) and aDSM. For example, some countries introduced the schedule of aDSM examinations proposed by the OR into their national clinical RR-TB protocols and standards. Examinations, including screening for COVID-19, hepatitis B and C virus (HBV and HCV), peripheral neuropathy, visual acuity and colour perception, were incorporated into the clinical patient monitoring schedule. Moreover, the severity assessment scale for AEs used in the research was introduced into the routine practice of TB programmes.^26^ A secondary finding of mSTR OR was that the prevalence of HCV seropositivity in participating countries was around 10%. Given that untreated HCV can lead to less successful treatment outcomes in patients with MDR/RR-TB and to aggravation of hepatotoxicity of TB drugs, the WHO Regional Office for Europe launched a multi-country OR initiative to introduce concomitant management of HCV and MDR/RR-TB, as the medicines currently prioritised by the WHO for management of MDR/RR-TB have no drug–drug interactions with directly acting agents used to treat HCV. This initiative is expected to improve clinical management for patients with co-infection, improve health outcomes and collect data for consideration by the WHO Consolidated Guidelines Development Group.

Last but not least, this OR provided equal access to shorter treatment for sub-populations often excluded from research initiatives, including prison inmates, pregnant women, children and people living with HIV with low CD4 count.

## STRENGTHS, WEAKNESSES AND NEXT STEPS

This is the largest observational cohort of MDR-RR/TB patients treated in 13 countries with the same care standards and approaches, with quality preparation, training and supervision. Data from a regional cohort of about 2,800 patients will allow analysis on effectiveness and safety of the mSTR, which were not previously available in the literature (albeit, without a comparator to the study regimens). External factors have had an impact on the provision of essential health services, including TB, which affected the follow-up of patients after treatment completion. However, the results of the main analysis will be published and followed by a stream of ancillary papers from the participating countries. Furthermore, this initiative has contributed to improving the OR capacity in the region and the development of additional OR in collaboration with existing networks. For example, 13 countries of the WHO European Region (Belarus, France, Italy, Lithuania, Netherlands (Kingdom of the), Portugal, Romania, Russian Federation, Serbia, Slovakia, Spain and United Kingdom) actively contributed to the Global Tuberculosis Network TB/COVID cohort^20–23^ and 15 (Belarus, Belgium, Bulgaria, Greece, Italy, Latvia, Lithuania, Netherlands (Kingdom of the), Portugal, Russian Federation, Slovakia, Spain, Sweden, Switzerland and United Kingdom) on the feasibility of aDSM at national level.^27,28^

## CONCLUSIONS

The mSTR OR initiative played a pivotal role in offering to patients a shorter regimen not previously available while enhancing treatment outcomes in 13 countries of the WHO European Region. This initiative has contributed to improving treatment monitoring procedures and clinical skills, providing substantial benefits to all current and future patients with RR/MDR-TB. Furthermore, the initiative has helped NTPs to identify and correct existing programmatic gaps. The enduring impact of the regional initiative is reflected in the establishment of the VMC and its webinars, providing a dedicated platform for case discussions and contributing to ongoing medical education. Furthermore, the project has cultivated a network of researchers, involving NTPs, clinicians and academia. This collaborative network is now equipped with the capacity to address and bridge regional research gaps initially identified by the ERA-TB, along with the pursuit of various research projects. A significant achievement lies in the project’s contribution to improving surveillance, data collection and analytical capabilities. This enhancement facilitates quality retrospective research but also allows for the utilisation of existing data even in the absence of dedicated funding, considering as conditions of OR are close to routine programmatic practices.^20–23,26‒28^ We anticipate that the experience gained through the mSTR OR regional initiative will serve as an inspiration for similar endeavours worldwide. The aim is to benefit people affected by TB by replicating successful models and approaches, fostering collaboration, and ultimately, contributing to global efforts in TB prevention, treatment and research.

## Footnotes

* Patients who have been treated with a long regimen composed of mSTR drugs within their current TB treatment episode and for whom results of fluoroquinolone sensitivity arrived >1 months from treatment initiation date were considered for inclusion in the OR upon consultation with the Virtual Medical Consilium (VMC) of the WHO Regional Office for Europe.
